# The association of body weight status with hypertriglyceridemia among community-dwelling older adults in regional China

**DOI:** 10.3389/fnut.2026.1985854

**Published:** 2026-10-27

**Authors:** Siyuan Lu, Yi Lu, Guofeng Ao, Luyan Yang, Ying Ji, Ting Xu, Wei Tang, Fei Xu

**Affiliations:** 1 School of Management, Xuzhou Medical University, Xuzhou, China; 2 First Clinical Medical College, Xuzhou Medical University, Xuzhou, China; 3 Nanjing Municipal Center for Disease Control and Prevention, Nanjing, China; 4 Nanjing Medical University School of Public Health, Nanjing, China; 5 Jiangsu Province Official Hospital, Nanjing, China; 6 Geriatric Hospital of Nanjing Medical University, Nanjing, China

**Keywords:** body mass index, Chinese, hypertriglyceridemia, obesity, older adults, overweight, triglyceride, waist circumstance

## Abstract

**Aim:**

To investigate associations of body weight with hypertriglyceridemia (HTG) among older adults in regional China.

**Methods:**

In this cross-sectional study, non-diabetic community-dwelling adults aged 60 + years and not receiving anti-lipid medications were randomly determined from Nanjing municipality, China, in 2023. HTG was defined based on fasting triglyceride concentration. Body weight status was assessed with waist circumstance (WC) and body mass index (BMI), respectively. Mixed-effects logistic regression analyses were conducted to compute odds ratios (ORs) and 95% confidence intervals (CIs) for examining associations of WC-based and BMI-based body weight status with HTG.

**Results:**

The prevalence of HTG was 29.3% among the overall 19,668 participants. Moreover, it was 25.3 and 32.3% among those without and with WC-based obesity, separately, and 22.6%, 33.1 and 39.9% for participants with normal BMI-based weight, overweight and obesity, respectively. Compared to their corresponding counterparts with normal weight, participants with WC-based obesity (OR = 1.37, 95%CI = 1.28, 1.44), BMI-based overweight (OR = 1.66, 95%CI = 1.55, 1.78) and obesity (OR = 2.14, 95%CI = 1.95, 2.35) were each more likely to experience HTG after adjustment for potential confounders. Additionally, when looking at the combined categories of WC- and BMI-based weight status, the positive associations between body weight status and HTG were also observed, with an exception of that the statistical estimate was not significant for subjects with WC-based obesity and normal BMI-based weight. Meaningfully, the scenarios of relationships between body weight and HTG among age−/sex-stratified participants were similar to those in overall subjects.

**Conclusion:**

Either WC-based weight, BMI-based weight, or their combined status was positively associated with HTG among the study older adults in regional China. This study has important health implications, suggesting that specific WC- or/and BMI-based body weight status shall be considered in clinical practice or public health campaigns for population-level effective HTG management.

## Introduction

Hypertriglyceridemia (HTG), a condition of elevated triglyceride (TG) concentration, has been well established as an independent risk factor of dysregulation in glucose metabolism and type 2 diabetes (T2D) (1–7). Moreover, diabetic patients, especially the older individuals, are usually recommended to have TG concentration under control for the purpose to reduce the risk of subsequent cardiovascular diseases in clinical practice (8–10). Definitely, TG control is a critical issue for both non-diabetic and diabetic adults (1–10). From the perspective of public health, TG control among non-diabetic people is of particular significance that it can reduce the risk of developing diabetes and its complications (1–7).

For prevention of HTG, it is crucial to identify the associated factors and then implement precise interventions (11). Excess body weight (overweight or obesity) is one of the identified modifiable risk factors of HTG (12–17). Moreover, both HTG and excess body weight, two common unfavorable health-related conditions in older adults, are collectively associated with metabolic disorders (8, 18). Meanwhile, a rapid process of population aging has been occurring worldwide (19), particularly in China (20). However, although excess body weight and HTG are age-dependent chronic conditions (21, 22), very few studies regarding the association between them are available specifically from older adults, especially those representative community-dwelling residents (12–17).

It is of important public health significance for building healthy-aging societies to investigate the relationship between excess body weight and HTG among older community-dwelling adults. Typically, diabetic patients are recommended to modify their lifestyle/behaviors or/and take medications for lipid control (8–10). Obviously, individuals with diabetes or receiving medications for lipid control are not appropriate for investigating the association of excess body weight with HTG, as their blood triglyceride levels may be intentionally modified and not reflected the “true state” without interference. Therefore, this study aimed to investigate the prevalence of HTG and its association with body weight status among older people without diabetes and not receiving medications for lipid control in Nanjing municipality of China.

## Methods

### Study design and participants

Data analyzed in this study were derived from the “Healthy Aging, Healthy Elders (HAHE) study”, a cross-sectional investigation conducted among representative community-residing adults aged 60 years or above in Nanjing municipality, China, in 2023 (23). Nanjing is a typical megacity in eastern China, having 12 administrative districts and 9.5 million registered residents (22.0% aged 60 + years) in 2023 (24). The study design, participant’s inclusion criteria and selection have been described in detail elsewhere (23). In brief, the HAHE-study was developed to assess the prevalence of common chronic conditions (e.g., diabetes, hypertension, excess body weight, abnormal lipid profiles) and associated factors among community-dwelling older adults. Eligible participants in HAHE-study referred to those: (1) registered community-dwelling residents in Nanjing municipality; (2) aged 60 + years; and (3) without psychiatric, or/and literacy-related problems. For the present study, participants who were experiencing diabetes or receiving medications for lipid control were excluded from the analysis.

The sample size of approximately 30,000 participants was estimated for the HAHE-study (23). Initially, 35,071 eligible participants were selected. Actually, 32,735 were successfully recruited (response rate = 93.3%). Among these recruited participants, 5,966 were diabetic patients and subsequently excluded, resulting in 26,769 older adults without diabetes. Then, of all the non-diabetic individuals, 7,101 self-reported receiving medications for lipid control and were also excluded. Finally, 19,668 eligible participants were analyzed in the present study. Such a large-scale sample size was sufficient for examining the relationship between excess body weight and HTG among participants. The selection flowchart of participants included in this study was shown in Figure 1.

**Figure 1:**
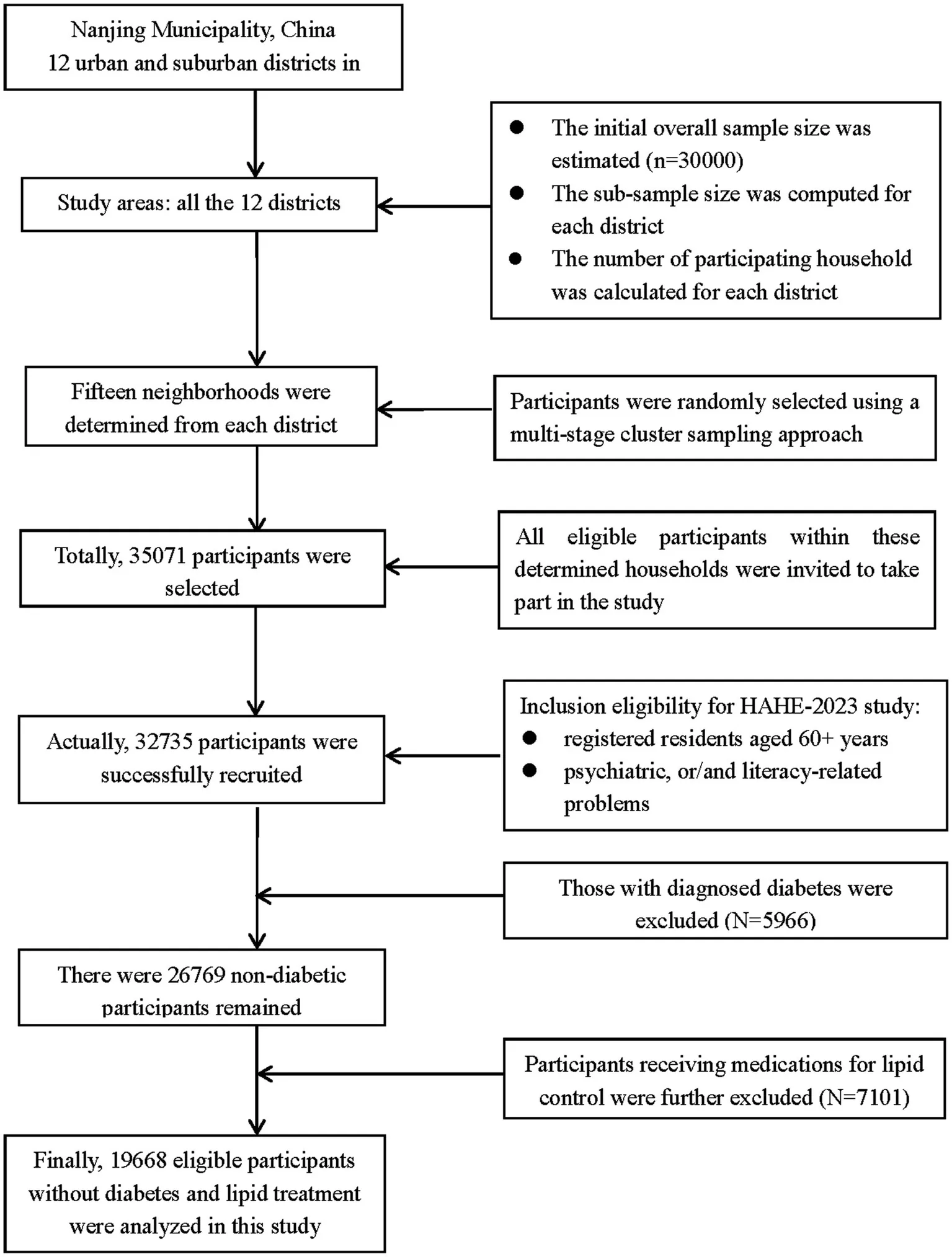
The selection flowchart of participants included in the study.

A multi-stage random sampling approach was employed to select participants from all the 12 urban and suburban districts in Nanjing in mid-2023 (23). First, each of the 12 districts was assigned with a sub-sample size according to the specific district-municipality proportion of older residents and estimated overall sample size. Moreover, based on an assumption that two older residents would be eligible to take part in the study within each household, the number of participating households was computed for each district. Second, 15 neighborhoods were randomly determined from each district. Third, participating households were chosen from each neighborhood. Finally, eligible participants within determined households were invited to take part in the HAHE-study.

All participants provided written informed consent before the survey. The original data collection protocol was reviewed and approved by the Ethics Committee of Nanjing Municipal Center for Disease Prevention and Control. Methods employed in this study were in line with the principles recommended in the Declaration of Helsinki.

### Data collection

In 2018, to warrant the comparability of data collected in studies across the whole country, Chinese Center for Non-communicable Disease Control and Prevention (CCNDC) released *The Scheme of Chinese Chronic Non-Communicable Disease (NCD) and Risk Factor Surveillance* (25). In this *scheme*, CCNDC recommended a standardized procedure for data collection, measurement instruments, and variable definition and classification to guide population investigations regarding common NCDs and associated factors (25). According to these recommendations, information on participant’s socio-demographic attributes, behaviors and lifestyle, history of common NCDs, and family history of diabetes were gathered via participant’s self-report using interviewer-administered questionnaire, while body weight and height, blood pressure (BP), and TG level were objectively assessed by research team members (25).

Each participant was measured twice to record weight to the nearest 0.1 kilograms and height to the nearest 0.01 meters. The mean values were used to compute body mass index (BMI) by dividing weight (kilogram) by height squared (meter^2^) (25). With a non-elastic flexible tape, waist circumference (WC) was also assessed twice to the nearest 0.1 centimeters at the midpoint between costal inferior and iliac crest for participants (25). Of course, mean WC readings were used to estimate the status of abdominal obesity for participants. For BP measurement, it was determined based on Korotkoff sounds with a sphygmomanometer (26). Moreover, BP was assessed at least two times and the mean value was recorded for analysis (25). Additionally, each participant’s fasting venous TG concentration was measured at a designated laboratory (25).

### Study variables

#### Outcome variable

The outcome event of interest was TG status. Based on fasting TG concentration, participants were categorized as having “normal TG (<1.7mmol/L)” or “HTG (≥1.7 mmol/L)” according to the cutoff value recommended for defining elevated TG levels in Chinese adults (13).

#### Independent variables

The independent variables were body weight status, including WC-based and BMI-based body weight status as well as their combinations. WC-based body weight status was categorized as “normal WC-based weight (WC < 90 cm for men, <85 cm for women)” or “WC-based obesity (WC ≥ 90 cm for men, ≥85 cm for women)” according to Chinese-sensitive sex-specific WC cutoff values, while BMI-based body weight status was classified as “normal BMI-based weight (BMI < 24.0)”, “BMI-based overweight (BMI = 24.0-27.9)”, or “BMI-based obesity (BMI ≥ 28.0)” according to BMI cutoffs recommended for Chinese adults (25).

In addition to investigating the separate associations of WC-based and BMI-based body weight status with HTG, their joint associations with HTG were also examined. For this purpose, participants were classified into six subgroups: “normal WC-based weight + normal BMI-based weight (the reference group)”, “normal WC-based weight + BMI-based overweight”, “normal WC-based weight + BMI-based obesity”, “WC-based obesity + normal BMI-based weight”, “WC-based obesity + BMI-based overweight”, “WC-based obesity + BMI-based obesity (the highest risk group)”.

#### Covariates

Covariates considered in multivariate regression analysis included: socio-demographic attributes (age, sex, residential location, educational attainment, marital status, healthcare insurance), behaviors and lifestyle (physical activity, sedentary behavior, smoking, drinking, meat and vegetable consumption), and hypertension. Specifically, age was classified as “60–69 [the young-old]”, “70–79 [the middle-old]” or “80+ [the oldest-old] years”, sex as “men” or “women”, residential location as “urban” or “suburban area”, educational level as “≤ 6”, “7–12” or “13+ years of schooling”, marital status as “single” or “with a spouse/partner (non-single)”, and healthcare insurance as “resident medical insurance (RMI)” or “employee medical insurance (EMI)” (25).

Definitions and classifications of behaviors and lifestyle were also adopted from the *Scheme of Chinese NCD Surveillance* (25). Physical activity (PA) and sedentary behavior (SB) were assessed with the validated Chinese version of the International Physical Activity Questionnaire (27, 28). PA level was categorized as “sufficient (≥ 150 min/week)” or “insufficient (< 150 min/week)” according to the sum of moderate PA time and doubled vigorous PA time in last week (29). SB, indicated with screen viewing time, was grouped as “prolonged (≥2 hours/day)” or “shortened (<2 hours/day)” (29). Moreover, alcohol drinking and cigarette smoking status were categorized as “drinker” or “non-drinker,” and “smoker” or “non-smoker,” respectively (25). Additionally, meat and vegetable consumption was measured with a validated Chinese food frequency questionnaire, and consumption frequencies in last week were recorded (30). Then, according to specific recommendations of weekly consumption frequency for Chinese older individuals by the Chinese Nutrition Society (31), participants were categorized as “recommendation reached (Yes)” or “recommendation not reached (No)” for meat and vegetable consumption, respectively.

Blood pressure status was defined based on the Chinese Guidelines for Blood Pressure Measurement (32). A hypertensive individual determined as an adult that he/she self-reported experiencing physician-diagnosed high blood pressure or his/her systolic/diastolic blood pressure was assessed ≥140/90 mmHg in the field survey (32). Otherwise, the participant was categorized as non-hypertensive individual in the analysis (32).

### Data analysis

Descriptive analysis was conducted to examine the difference in body weight status and fasting triglyceride level by selected socio-demographic attribute. Next, two mixed-effects logistic regression models were introduced to compute odds ratios (ORs) and 95% confidence intervals (CIs) for assessing associations of body weight status with HTG among older adults. Model 1 was an unadjusted analysis with body weight status as the single independent variable and adjustment for community-level clustering effect. Model 2 was a multi-factor analysis with adjustment for age (where applicable), sex (where applicable), urban/rural residence, educational attainment, marital status, healthcare insurance, PA, SB, smoking, drinking, meat and vegetable consumption, hypertension in addition to those included in Model 1. The *p* < 0.05 (two-sided) was treated as statistically significant. Data were analyzed with R (version 4.4.3, R Foundation for Statistical Computing, Austria).

## Results

Totally, 19,668 non-diabetic older adults who did not receive medications for lipid management were included in the analysis. Of them, 49.5% were male and 55.5% were urban residents. There were 67.7 and 6.1% aged 60–69 and 80 + years, respectively, while 40.9 and 9.3% attained education of 6- and 13 + years of schooling. Moreover, 14.5% were single individuals (Table 1).

**Table 1:** Selected socio-demographic characteristics of study participants in Nanjing, China (*N* = 19,668)^*^.

Characteristic	Participants (*n*, %)		
	Overall	Men	Women
Total	19,668	9,742 (49.5)	9,926 (50.5)
Age (years)			
60–69	13,309 (67.7)	6,487 (66.6)	6,822 (68.7)
70–79	5,158 (26.2)	2,651 (27.2)	2,507 (25.3)
80+	1,201 (6.1)	604 (6.2)	597 (6.0)
Residential location			
Urban	10,913 (55.5)	5,361 (55.0)	5,552 (55.9)
Suburban	8,755 (44.5)	4,381 (45.0)	4,374 (44.1)
Educational attainment (years of schooling)			
6-	8,047 (40.9)	3,090 (31.7)	4,957 (49.9)
7–12	9,804 (49.8)	5,494 (56.4)	4,310 (43.4)
13+	1817 (9.3)	1,158 (11.9)	659 (6.7)
Marital status			
Single	2,846 (14.5)	1,104 (11.3)	1742 (17.5)
Having a spouse/partner	16,822 (85.5)	8,638 (88.7)	8,184 (82.5)
Healthcare insurance^¶^			
RMI	9,023 (45.9)	4,098 (42.1)	4,925 (49.6)
EMI	10,645 (54.1)	5,644 (57.9)	5,001 (50.4)

*Participants were older adults aged 60 + years who were not diabetic patients and without receiving lipid treatment.

^¶^RMI, resident basic medical insurance scheme; EMI, employee basic medical insurance scheme.

Table 2 presents the proportions of body weight status among participants by socio-demographic characteristics. The prevalence was 57.7% (95%CI = 57.0%, 58.4%) for WC-based obesity, 41.3% (95%CI = 40.6%, 42.0%) for BMI-based overweight, and 13.9% (95%CI = 13.4%, 14.4%) for BMI-based obesity among participants. Moreover, the prevalence of WC-based obesity was different across subgroups of age, sex, residential location, educational attainment, marital status or healthcare insurance, while the status of BMI-based body weight also significantly differed in categories of these factors with an exception of residential location.

**Table 2:** The distribution of body weight status by selected characteristics of study participants in Nanjing, China (*N* = 19,668)^*^.

Characteristic	Participants (*n*, %) ^#^									
	Overall	WC-based body weight status		*χ^2^*	*p* value ^§^	BMI-based body weight status			*χ^2^*	*p* value^§^
		Normal	Obese			Normal	Overweight	Obese		
Total	19,668	8,323 (42.3)	11,345 (57.7)			8,813 (44.8)	8,129 (41.3)	2,726 (13.9)		
Age (years)										
60–69	13,309 (67.7)	5,636 (42.3)	7,673 (57.7)	7.26	0.026	5,823 (43.8)	5,664 (42.6)	1822 (13.6)	42.25	<0.001
70–79	5,158 (26.2)	2,138 (41.5)	3,020 (58.5)			2,363 (45.8)	2044 (39.6)	751 (14.6)		
80+	1,201 (6.1)	549 (45.7)	652 (54.3)			627 (52.2)	421 (35.1)	153 (12.7)		
Sex										
Men	9,742 (49.5)	4,541 (46.6)	5,201 (53.4)	145.89	<0.001	4,289 (44.0)	4,269 (43.8)	1,184 (12.2)	72.15	<0.001
Women	9,926 (50.5)	3,782 (38.1)	6,144 (61.9)			4,524 (45.6)	3,860 (38.9)	1,542 (15.5)		
Residential location										
Urban	10,913 (55.5)	5,036 (46.1)	5,877 (53.9)	147.28	<0.001	4,862 (44.6)	4,579 (42.0)	1,472 (13.4)	5.14	0.076
Suburban	8,755 (44.5)	3,287 (37.5)	5,468 (62.5)			3,951 (45.2)	3,550 (40.5)	1,254 (14.3)		
Educational attainment (years of schooling)										
6-	8,047 (40.9)	3,534 (43.9)	4,513 (56.1)	14.74	0.001	3,512 (43.6)	3,259 (40.5)	1,276 (15.9)	69.93	<0.001
7–12	9,804 (49.8)	4,027 (41.1)	5,777 (58.9)			4,399 (44.8)	4,123 (42.1)	1,282 (13.1)		
13+	1817 (9.3)	762 (41.9)	1,055 (58.1)			902 (49.7)	747 (41.1)	168 (9.2)		
Marital status										
Single	2,846 (14.5)	1,462 (51.4)	1,384 (48.6)	111.72	<0.001	1,337 (47.0)	1,110 (39.0)	399 (14.0)	7.92	0.019
Having a spouse/partner	16,822 (85.5)	6,861 (40.8)	9,961 (59.2)			7,476 (44.5)	7,019 (41.7)	2,327 (13.8)		
Healthcare insurance^¶^										
RMI	9,023 (45.9)	3,685 (40.8)	5,338 (59.2)	14.91	<0.001	4,056 (45.0)	3,653 (40.4)	1,314 (14.6)	8.90	0.012
EMI	10,645 (54.1)	4,638 (34.6)	6,007 (56.4)			4,757 (44.7)	4,476 (42.0)	1,412 (13.3)		

*Participants were older adults aged 60 + years who were not diabetic patients and without receiving lipid treatment.

^#^Body weight status was defined as “normal WC-based weight (WC < 90 cm for men, <85 cm for women) or WC-based obesity (WC ≥ 90 cm for men, ≥85 cm for women)” based on sex-specific waist circumference (WC), or “normal BMI-based weight (BMI < 24.0), BMI-based overweight (BMI = 24.0, 27.9), or BMI-based obesity (BMI ≥ 28.0)” based on body mass index (BMI) according to recommendations for Chinese adults.

^§^Chi-square test.

^¶^RMI, resident medical insurance scheme; EMI, employee medical insurance scheme.

Table 3 displays the prevalence of HTG among participants in the study. Overall, the prevalence of HTG was 29.3% (95%CI = 28.7%, 30.0%) among community-dwelling older adults in regional China. Moreover, HTG prevalence decreased with age (30.5% *vs*. 27.8% *vs*. 23.5%: the younger-old *vs*. middle-old *vs*. the oldest-old), while it was higher among women (women *vs*. men: 31.7% *vs*. 26.9%) or suburban individuals (sub-urban *vs*. urban: 30.3% *vs*. 28.6%) relative to the corresponding counterparts. However, no difference of HTG prevalence was observed in participants by educational level, marital status, or healthcare insurance.

**Table 3:** The distribution of fasting triglyceride level by selected characteristics of study participants in Nanjing, China (*N* = 19,668)^*^.

Characteristic	Participants (*n*, %) ^#^			*χ^2^*	*p* value ^§^
	Overall	Normal level of triglyceride	HTG		
Total	19,668	13,897 (70.7)	5,771 (29.3)		
Age (years)					
60–69	13,309 (67.7)	9,255 (69.5)	4,054 (30.5)	33.69	<0.01
70–79	5,158 (26.2)	3,723 (72.2)	1,435 (27.8)		
80+	1,201 (6.1)	919 (76.5)	282 (23.5)		
Sex					
Men	9,742 (49.5)	7,126 (73.1)	2,616 (26.9)	57.69	<0.01
Women	9,926 (50.5)	6,771 (68.3)	3,155 (31.7)		
Residential location					
Urban	10,913 (55.5)	7,794 (71.4)	3,119 (28.6)	6.86	<0.01
Suburban	8,755 (44.5)	6,103 (69.7)	2,652 (30.3)		
Educational attainment (years of schooling)					
6-	8,047 (40.9)	5,692 (70.7)	2,355 (29.3)	0.50	0.78
7–12	9,804 (49.8)	6,910 (70.5)	2,894 (29.5)		
13+	1817 (9.3)	1,295 (71.3)	522 (28.7)		
Marital status					
Single	2,846 (14.5)	2008 (70.5)	838 (29.4)	0.02	0.90
Having a spouse/partner	16,822 (85.5)	11,889 (70.7)	4,933 (29.3)		
Healthcare insurance^¶^					
RMI	9,023 (45.9)	6,372 (70.6)	2,651 (29.4)	0.01	0.92
EMI	10,645 (54.1)	7,525 (70.7)	3,120 (29.3)		

*Participants were older adults aged 60 + years who were not diabetic patients and without receiving lipid treatment.

^#^Fasting triglyceride status was classified as normal (<1.7 mmol/L), or HTG (hypertriglyceridemia) (fasting triglyceride concentration ≥1.7) according to Chinese Guidelines for Lipid Management (2023).

^§^Chi-square test.

^¶^RMI, resident medical insurance scheme; EMI, employee medical insurance scheme.

Figure 2 shows the association of body weight status with HTG among overall participants. After adjustment for potential confounders, compared to those with normal WC-based weight, participants with WC-based obesity were more likely to experience HTG (adj. OR = 1.37, 95%CI = 1.28, 1.44). Moreover, subjects with BMI-based overweight or obesity were also at a significantly higher risk of HTG (adj. OR = 1.66, 95%CI = 1.55, 1.78; adj. OR = 2.14, 95%CI = 1.95, 2.35, separately) relative those with normal BMI-based weight. However, there was no interactions between WC-based and BMI-based weight status on HTG (*p* = 0.23 for WC-based obesity*BMI-based overweight; *p* = 0.63 for WC-based obesity*BMI-based obesity).

**Figure 2:**
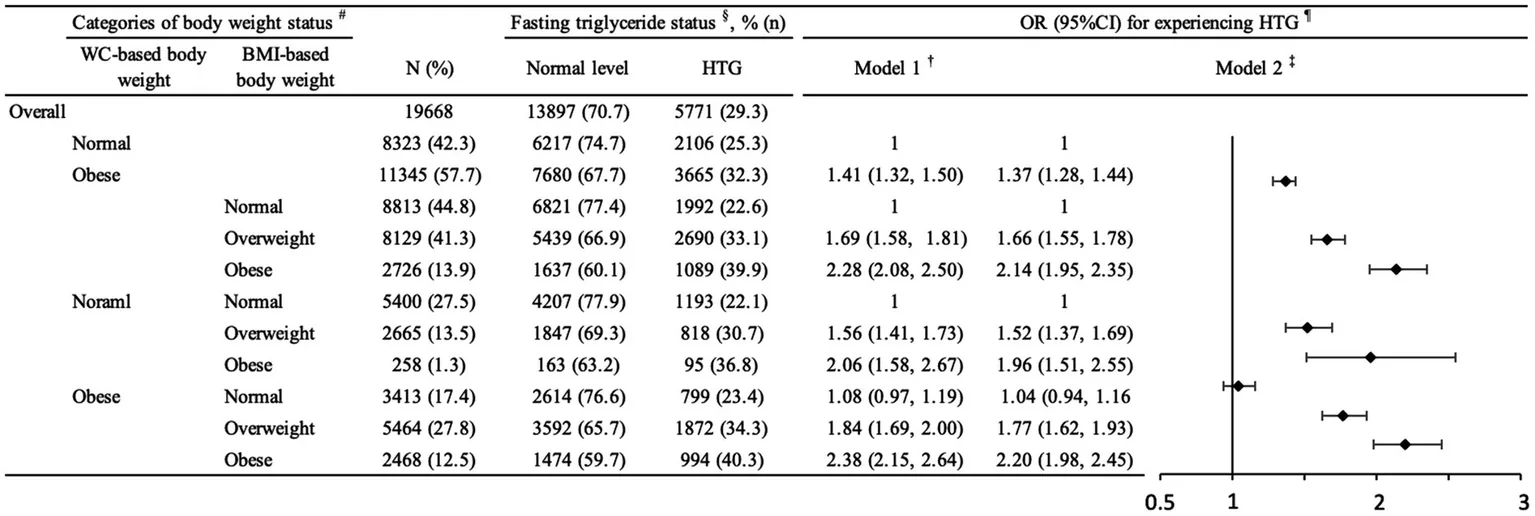
The separate and combined associations of central and general body weight with hypertriglyceridia with hypertriglyceridemia among overall participants in Nanjing, China (*N* = 19,668). *Participants were older adults aged 60 + years who were not diabetic patients and without receiving lipid treatment. ^#^Body weight status was defined as “normal WC-based weight (WC < 90 cm for men, <85 cm for women) or WC-based obesity (WC ≥ 90 cm for men, ≥85 cm for women)” based on sex-specific waist circumference (WC), or “normal BMI-based weight (BMI < 24.0), BMI-based overweight (BMI = 24.0, 27.9), or BMI-based obesity (BMI ≥ 28.0)” based on body mass index (BMI) according to recommendations for Chinese adults. ^§^Fasting triglyceride status was classified as normal (<1.7 mmol/L), or HTG (hypertriglyceridemia) (fasting triglyceride concentration ≥1.7) according to Chinese Guidelines for Lipid Management (2023). ^¶^OR, odds ratios; 95%CI, 95% confidence interval. ^†^Model 1 was a univariate mixed-effects logistic regression analysis with body weight status as the single predictor and adjustment for community-level clustering effect. ^‡^Model 2 was a multivariate mixed-effects logistic regression analysis with further adjustment for age, sex, urban/rural residence, educational attainment, marital status, healthcare insurance, physical activity, sedentary behavior, smoking, drinking, meat and vegetable consumption, hypertension in addition to those included in Model 1.

Additionally, after adjustment for potential influencing factors, compared to those with “normal WC-based weight + normal BMI-based weight”, participants with “normal WC-based weight + BMI-based overweight” (OR = 1.52, 95%CI = 1.37, 1.69), “normal WC-based weight + BMI-based obesity” (OR = 1.96, 95%CI = 1.51, 2.55), “WC-based obesity + BMI-based overweight” (OR = 1.77, 95%CI = 1.62, 1.93), or “WC-based obesity + BMI-based obesity” (OR = 2.20, 95%CI = 1.98, 2.45) were all at significantly higher odds for experiencing HTG, with an exception of non-significant relationship between “WC-based obesity + normal BMI-based weight” and HTG (OR = 1.04, 95%CI = 0.94, 1.16) (Figure 2).

Figures 3, 4 demonstrates the association of body weight status with HTG among participants by age and sex, separately. The separate relationship between WC-based weight and BMI-based weight with HTG among participants by age or sex was the same as that in overall subjects. Moreover, the scenarios of the associations of combined WC-based and BMI-based body weight status with HTG among participants stratified by age or sex were similar to those observed in overall subjects accordingly.

**Figure 3:**
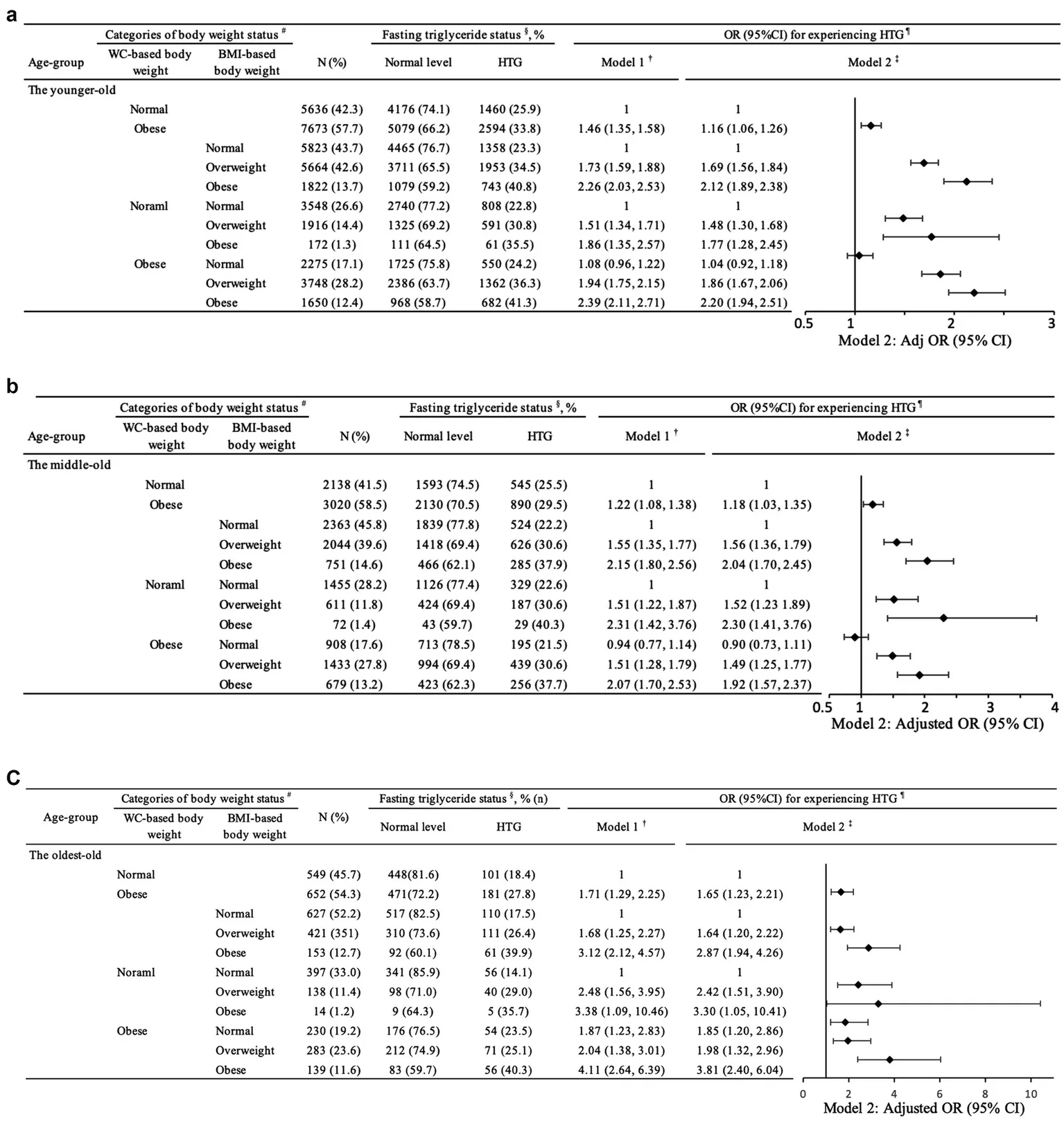
**(a)** The separate and combined associations of central and general body weight with hypertriglyceridia with hypertriglyceridemia among participants aged 60–69 years in Nanjing, China (*N* = 13,309). *Participants were older adults aged 60–69 years who were not diabetic patients and without receiving lipid treatment. **(b)** The separate and combined associations of central and general body weight with hypertriglyceridia with hypertriglyceridemia among participants aged 70–79 years in Nanjing, China (*N* = 5,158)^*^. *Participants were older adults aged 70–79 years who were not diabetic patients and without receiving lipid treatment. **(c)** The separate and combined associations of central and general body weight with hypertriglyceridia with hypertriglyceridemia among participants aged 80 + years in Nanjing, China (*N* = 1,201)^*^. *Participants were older adults aged 80 + years who were not diabetic patients and without receiving lipid treatment. ^#^Body weight status was defined as “normal WC-based weight (WC < 90 cm for men, <85 cm for women) or WC-based obesity (WC ≥ 90 cm for men, ≥85 cm for women)” based on sex-specific waist circumference (WC), or “normal BMI-based weight (BMI < 24.0), BMI-based overweight (BMI = 24.0, 27.9), or BMI-based obesity (BMI ≥ 28.0)” based on body mass index (BMI) according to recommendations for Chinese adults. ^§^Fasting triglyceride status was classified as normal (<1.7 mmol/L), or HTG (hypertriglyceridemia) (fasting triglyceride concentration ≥1.7) according to Chinese Guidelines for Lipid Management (2023). ^¶^OR, odds ratios; 95%CI, 95% confidence interval. ^†^Model 1 was a univariate mixed-effects logistic regression analysis with body weight status as the single predictor and adjustment for community-level clustering effect. ^‡^Model 2 was a multivariate mixed-effects logistic regression analysis with further adjustment for, sex, urban/rural residence, educational attainment, marital status, healthcare insurance, physical activity, sedentary behavior, smoking, drinking, meat and vegetable consumption, hypertension in addition to those included in Model 1.

**Figure 4:**
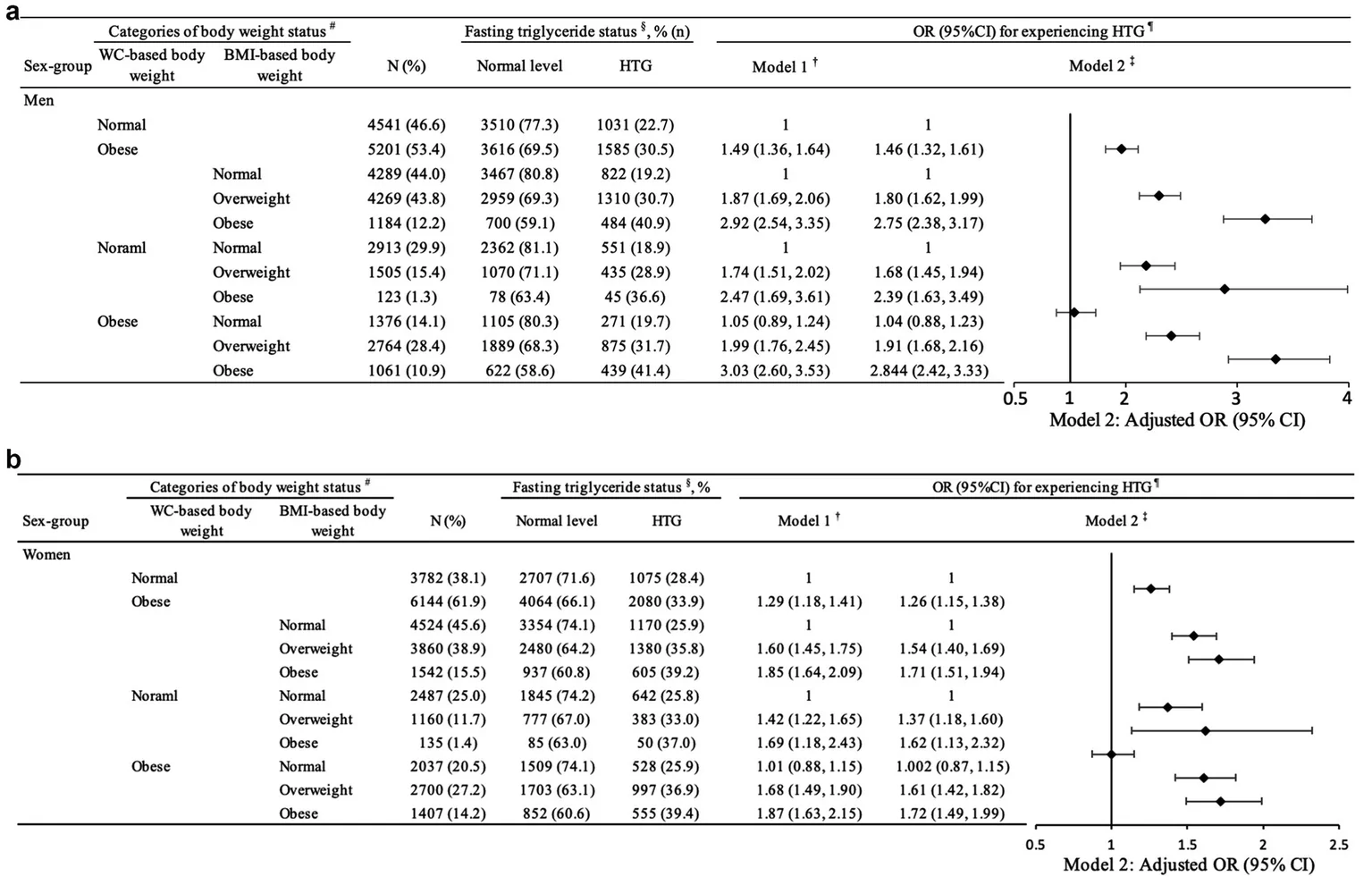
**(a)** The separate and combined associations of central and general body weight with hypertriglyceridia with hypertriglyceridemia among male participants in Nanjing, China (*N* = 9,742). *Participants were older adults aged 60 + years who were not diabetic patients and without receiving lipid treatment. **(b)** The separate and combined associations of central and general body weight with hypertriglyceridia with hypertriglyceridemia among female participants in Nanjing, China (*N* = 9,926). *Participants were older adults aged 60 + years who were not diabetic patients and without receiving lipid treatment. ^#^Body weight status was defined as “normal WC-based weight (WC < 90 cm for men, <85 cm for women) or WC-based obesity (WC ≥ 90 cm for men, ≥85 cm for women)” based on sex-specific waist circumference (WC), or “normal BMI-based weight (BMI < 24.0), BMI-based overweight (BMI = 24.0, 27.9), or BMI-based obesity (BMI ≥ 28.0)” based on body mass index (BMI) according to recommendations for Chinese adults. ^§^Fasting triglyceride status was classified as normal (<1.7 mmol/L), or HTG (hypertriglyceridemia) (fasting triglyceride concentration ≥1.7) according to Chinese Guidelines for Lipid Management (2023). ^¶^OR, odds ratios; 95%CI, 95% confidence interval. ^†^Model 1 was a univariate mixed-effects logistic regression analysis with body weight status as the single predictor and adjustment for community-level clustering effect. ^‡^Model 2 was a multivariate mixed-effects logistic regression analysis with further adjustment for age, urban/rural residence, educational attainment, marital status, healthcare insurance, physical activity, sedentary behavior, smoking, drinking, meat and vegetable consumption, hypertension in addition to those included in Model 1.

However, different from that non-significant association between “WC-based obesity + normal BMI-based weight” and HTG” was observed in overall participants, the oldest-old people with WC-based obesity and normal BMI-based weight were at a significantly higher risk to experience HTG (OR = 1.85, 95%CI = 1.20, 2.86) compared to their counterparts with normal WC-based and BMI-based weight. Additionally, when data on the oldest-older adults were further stratified by sex (not shown in Figures), it was examined that such a positive association of WC-based obesity and normal BMI-based weight with HTG existed within men (OR = 2.73, 95%CI = 1.37, 5.40) but not in women (OR = 1.38, 95%CI = 0.78, 2.44).

## Discussion

This large scale population study aimed to investigate the prevalence of body weight status and primarily examine the individual and combined associations of WC-based and BMI-based body weight status with HTG among community-dwelling older adults who were not diabetic patients and did not receive medications for lipid treatment in regional China. It was observed that the prevalence was 57.7% for WC-based obesity, 41.3% for BMI-based overweight and 13.9% for BMI-based obesity, while the prevalence of HTG was 29.3% among overall participants. Individually, either WC-based or BMI-based body weight status was positively associated with HTG among overall participants, each stratum of age or sex. Moreover, participants with the combined WC-based obesity and BMI-based excess body weight were also at higher odds to experience HTG compared to those simultaneously with normal WC-based and BMI-based body weight, with an exception of participants with WC-based obesity and normal BMI-based weight. The scenarios of joint associations of WC-based and BMI-based body weight status with HTG among participants stratified by age or sex were similar to those in overall subjects in this study.

For China, the most recent prevalence rates of WC-based obesity (41.5%), BMI-based general overweight (36.6%) and obesity (13.6%) among older adults were documented in *Report on the Health Status of Older Adults in China (2021)*, which was estimated based on nationally representative data collected among older adults aged 60 years and older in 2018 (33). Obviously, the prevalence rates of WC-based obesity (57.7% *vs*. 41.5%) and BMI-based overweight (41.3% *vs*. 36.6%) observed in this study based on data gathered in 2023 were higher than the corresponding figures recorded in the national 2018 survey (33). However, no significant difference (13.9% *vs*. 13.6%) existed in BMI-based obesity prevalence between these two studies (33). Moreover, the prevalence of HTG observed in this study was significantly higher than that recorded in the most recent national survey (29.3% *vs*. 17.1%) (33). The discrepancies may be due to that different participants were involved in this study and the national survey (33). In this study, the participants were those community-dwelling non-diabetic older adults who did not receive medications for lipid treatment, while the subjects in the national survey were overall community-dwelling older adults (33). Particularly, the differences may also be, at least partly, explained by that Nanjing municipality is an economically-developed and well-urbanized city in China (33). The findings highlight that the burden of WC-based obesity, BMI-based excess bodyweight, and HTG was substantially heavy for older adults without diabetes in Nanjing of China.

It was examined that either WC-based obesity, BMI-based overweight or obesity was positively associated with HTG in this study. This was in line with the previous reports (12–17). When looking at the combinations of WC-based and BMI-based body weight status, it is notable that, compared to subjects with both normal WC- and BMI-based body weight status simultaneously, those with normal WC-based weight, and either BMI-based overweight or obesity were more likely to experience HTG, while participants with normal BMI-based body weight and WC-based obesity were at the similar level of risk for experiencing HTG. It suggests that BMI-based body weight may be a more sensitive factor of HTG than WC-based weight for older adults without diabetes, particularly considering that no interaction of WC-based and BMI-based body weight on HTG was examined in this study. Additionally, the scenarios of associations of WC-based and BMI-based weight with HTG are similar among overall participants, each sub-group of sex or age, implying that such an association between body weight and HTG may hold for general community-dwelling older adults who are non-diabetic individuals and not receiving medications for lipid management in regional China. However, the findings in this study are not consistent with those reported in previous studies among adults in Taiwan of China or Korea (34, 35). This may be due to that participants in these two studies had different characteristics from the counterparts in the present one (34, 35). In the Taiwan and Korean studies, subjects experiencing diabetes or using anti-lipid medications were not excluded from analysis, and were younger with the age of 35–64 and 19–64 years, respectively (34, 35).

Unfortunately, it is not easy to explain why WC-based obesity alone was not but BMI-based excess body weight alone was positively associated with HTG when the condition of normal WC-based and BMI-based body weight was used as the reference in this study, as, usually, it is accepted that WC-based obesity is an independent and strong predictor of HTG (34, 35). One plausible reason may be that WC-based obese and BMI-based excess body weight were not differentiated from each other, implying that participants with WC-based obesity might be with BMI-based excess body weight simultaneously in previous studies (34, 35). For further understanding the individual and combined association of WC-based and BMI-based body weight status with HTG among older adults, longitudinal studies are encouraged to explore the potential causality between them. Importantly, to elucidate the “true single” association of WC-based and BMI-based body weight with HTG is critical for clinical and public health practice against HTG.

Strengths in this study are worthy of mention. First, the community-dwelling participants were randomly chosen from all the 12 districts of Nanjing municipality, with a sample size of nearly 20,000, warranting a sufficient statistical power. Second, those older residents with diabetes or medications for lipid treatment were excluded from analysis, implying that TG level was not intentionally modified due to diabetes and/or lipid management. Third, weight, height, and TG were objectively assessed using standardized approaches (25). Fourth, classical HTG-associated influencing factors — socio-demographic characteristics, lifestyle and behaviors, and hypertension — were controlled for in the multivariate analysis. Fifth, individual and combined associations of WC-based and BMI-based body weight status with HTG were examined simultaneously in a single study. The last, the same scenarios of associations between body weight and HTG were found not only within overall participants but also among each stratum of sex or age, suggesting that the findings may hold for general community-dwelling older residents in the study municipality.

This study also has several limitations. First, no causality can be inferred for the relationship between body weight and HTG in this cross-sectional study. Second, data on socio-demographic characteristics, lifestyle, behaviors, and hypertension were self-reported, implying that potential recall bias might exist. Third, due to the skewed distribution, meat and vegetable consumption frequencies were treated as binary measures in the analysis based on recommendations by Chinese Nutrition Society (31). This implies that the potential influence of them, particularly meat, on HTG might not be fully captured, although they were not the primary explanatory variables but just covariates adjusted for in analysis. Fourth, hypertensive patients were included in the study, while it is well-known that certain anti-hypertensive medications can inherently elevate triglyceride levels. However, due to no data available on specific anti-hypertensive medications, we could not have it considered in multivariate analysis. In future, longitudinal or intervention studies are encouraged to investigate the potential causal relationship between body weight and HTG for older adults by different combinations of WC-based and BMI-based weight status, as it is really critical for precise HTG intervention through personalized excess body weight control.

In conclusion, over half of participants experienced either WC-based obesity or BMI-based excess body weight. Moreover, either WC-based or BMI-based body weight was positively associated with HTG among community-dwelling non-diabetic older adults who did not receive medications for lipid control in China. Additionally, BMI-based excess body weight may be associated with HTG more strongly compared to WC-based obesity. This study has important clinical and public health implications. For clinical practice, it may be of help for effective HTG management when doctors write tailored prescriptions with specific consideration of patients’ WC-based or/and BMI-based body weight. In the context of public health campaigns, it is also necessary to put into consideration of specific WC-based or BMI-based body weight for developing population-level HTG-related health promotion programs targeting older adults.

## Data Availability

The original contributions presented in the study are included in the article/supplementary material, further inquiries can be directed to the corresponding author.
